# *Mycobacterium abscessus* and Children with Cystic Fibrosis

**DOI:** 10.3201/eid0912.020774

**Published:** 2003-12

**Authors:** Isabelle Sermet-Gaudelus, Muriel Le Bourgeois, Catherine Pierre-Audigier, Catherine Offredo, Didier Guillemot, Sophie Halley, Chantal Akoua-Koffi, Véronique Vincent, Valérie Sivadon-Tardy, Agnès Ferroni, Patrick Berche, Pierre Scheinmann, Gérard Lenoir, Jean-Louis Gaillard

**Affiliations:** * Hôpital Necker-Enfants Malades, Assistance Publique–Hôpitaux de Paris, Paris, France; † Institut Pasteur, Paris, France; ‡ Hôpital Raymond Poincaré, Assistance Publique–Hôpitaux de Paris, Garches, France

**Keywords:** Cystic fibrosis, *Mycobacterium abscessus*, nontuberculous mycobacteria, *hsp65* sequencing, pulsed-field gel electrophoresis, epidemiology

## Abstract

We prospectively studied 298 patients with cystic fibrosis (mean age 11.3 years; range 2 months to 32 years; sex ratio, 0.47) for nontuberculous mycobacteria in respiratory samples from January 1, 1996, to December 31, 1999. *Mycobacterium abscessus* was by far the most prevalent nontuberculous mycobacterium: 15 patients (6 male, 9 female; mean age 11.9 years; range 2.5–22 years) had at least one positive sample for this microorganism (versus 6 patients positive for *M. avium* complex), including 10 with >3 positive samples (versus 3 patients for *M. avium* complex). The *M. abscessus* isolates from 14 patients were typed by pulsed-field gel electrophoresis: each of the 14 patients harbored a unique strain, ruling out a common environmental reservoir or person-to-person transmission. Water samples collected in the cystic fibrosis center were negative for *M. abscessus*. This major mycobacterial pathogen in children and teenagers with cystic fibrosis does not appear to be acquired nosocomially.

Since 1990, an increasing number of studies have reported the recovery of nontuberculous mycobacteria from the respiratory tract of patients with cystic fibrosis (*1*–*4*). *Mycobacterium abscessus* (formerly *M. chelonae* subsp. *abscessus*), a rapidly growing mycobacterium of the *M. fortuitum* complex, is of particular concern. It can cause severe lung disease, which spontaneously advances until it becomes debilitating or fatal (*5*,*6*). It may be responsible for disseminated infections in patients undergoing lung transplantation (*7*). This organism is usually also susceptible to only a few drugs (*8*), and some strains may exhibit multidrug resistance (*7*).

The frequency of isolation of *M. abscessus* in cystic fibrosis patients is unclear. Many studies on nontuberculous mycobacteria in such patients did not distinguish *M. abscessus* and *M. chelonae*, formerly two subspecies of *M. chelonae*, and used the designation *M. chelonae–M. abscessus*, *M. chelonae* group, or even *M. fortuitum* complex. Moreover, most studies were conducted with adults (*1*,*2*,*4*). How cystic fibrosis patients become contaminated is also poorly understood. *M. abscessus* has been reported to be acquired iatrogenically in non–cystic fibrosis patients (*9*). The members of the *M. fortuitum* complex are saprophytic organisms living in soil and water that are ubiquitous in hospital environments and survive well in adverse conditions (*10*–*13*). Aerosols, pulmonary function equipment, and bronchoscopes are thus potential sources of contamination for patients with cystic fibrosis. Alternatively, transmission from patients to patients attending the same department-care facilities might occur, although this finding has been recently challenged (*14*).

We encountered one case of *M. abscessus* infection in a patient with cystic fibrosis in 1995. The recovery of this unusual pathogen prompted us to prospectively evaluate the rate of isolation of *M. abscessus* in the cystic fibrosis patients attending our center, the degree of transmissibility of this organism, and its clonality, by using DNA-based identification and typing systems.

## Patients and Methods

### Description of Study

All patients with cystic fibrosis who attended the pediatric department of Necker Hospital for Sick Children from January 1, 1996, to December 31, 1999, provided at least one sputum sample per year, which was processed for the culture of acid-fast bacilli (AFB). Patients who provided a positive sample then submitted >3 sputum samples for AFB smear and culture over the next 3 months. AFB smears and cultures were checked quarterly thereafter.

### Cultures of Respiratory Specimens

Specimens were decontaminated with NALC-NaOH-oxalic acid (0.25% *N*-acetyl-L-cysteine–1% sodium hydroxide–5% oxalic acid) (*15*). AFB smears were stained with auramine-rhodamine and scored as previously described (*16*). Two Löwenstein-Jensen slants were inoculated for each specimen, one of which was incubated at 37°C and the other at 30°C. The slants were examined twice weekly for 2 weeks and then weekly for a further 10 weeks.

### Environmental Samples

Water samples taken from the hospital’s hot and cold water supply systems were collected in sterile plastic bottles. Samples (100 mL) were decontaminated with 1% NaOH without prior concentration by filtration (*17*). The inner surfaces of respiratory devices (e.g., nebulizers, bronchoscopes) were rinsed with 1 to 10 mL of sterile distilled water; the water used for rinsing was processed for the culture of AFB without prior decontamination with 1% NaOH.

### Species Identification

Rapidly growing mycobacteria recovered from clinical and environmental samples were identified by standard techniques (*17*) and *hsp65* sequencing (*18*). The *M. avium* complex was identified by the AccuProbe technique (Gen-Probe Inc., San Diego, CA). The *hsp65* genomovars of *M. abscessus* were referred to as T (identical to the type-strain *M. abscessus* ATCC 19977^T^), -5a (differing from ATCC 19977^T^ by 5 nt, and identical to the reference strain *M. abscessus* IP970272), -5b (differing from ATCC 19977^T^ by 5 nt, and identical to the reference strain *M. abscessus* IP970453), and –6 (differing from ATCC 19977^T^ by 6 nt, and identical to the reference strain *M. abscessus* IP140420009), as previously described (*18*).

### PFGE Analysis

*M. abscessus* isolates were analyzed by PFGE as described by Wallace et al., with minor modifications (*9*). Restriction fragments obtained after digestion with *Dra*I and *Xba*I were separated in 0.5 x TBE buffer (0.025 M Tris, 0.5 mM EDTA, and 0.025 M boric acid) supplemented with 50 μM thiourea (*19*), using a CHEF-DR III system (Bio-Rad, Richmond, CA) at 14°C and 6 V/cm^2^. Pulse times were ramped linearly from 1.5 to 21.5 s for 23 h. A size standard (bacteriophage λ concatemers) was run in parallel in each experiment. Restriction patterns were analyzed with the Taxotron package (Taxolab Software, Institut Pasteur, Paris, France) comprising the RestrictoScan, RestrictoTyper, Adanson, and Dendrograph programs.

## Results

### Screening the Study Population for Nontuberculous Mycobacteria

A total of 298 patients with cystic fibrosis (1,525 sputum samples; mean of 5.0 samples per patient) followed up at our institution were screened for *M. abscessus* from January l, 1996, to December 31, 1999. The age of the patients ranged from 2 months to 32 years (mean 11.3 years). The sex ratio was 0.47 (140 male/158 female patients). Samples from two patients could not be analyzed because the samples were repeatedly contaminated (<1 interpretable culture per year during the study period). Of the 296 patients with interpretable cultures, 29 (9.80%) provided at least one sample positive for nontuberculous mycobacteria. Twelve of the 296 patients had *M. abscessus* alone, 3 had *M. abscessus* and *M. gordonae*, 6 had *M. avium* complex, 4 had *M. gordonae*, 1 had *M. fortuitum*, 1 had *M. kansasii*, and 2 had organisms not related to any known species. Thirteen patients provided at least three positive samples, 10 involving *M. abscessus* and 3 *M. avium* complex. Two of these patients were twin sisters, one colonized with *M. abscessus* (patient no. 5) and the other with *M. avium* complex.

### Description of Cases with *M. abscessus* Isolation

Fifteen (5%) of the 296 patients with interpretable cultures provided at least one sample positive for *M. abscessus*. Ten of these patients had >3 positive samples, including six with positive AFB smears (Table). Mycobacterial disease was documented in four patients: a 16-year-old boy (patient no. 4) with parenchymal condensation of the left lower lobe on chest x-ray and computed tomographic (CT) scan, which disappeared only under anti–*M. abscessus* treatment; a 10-year-old boy (patient no. 6), whose rapidly deteriorating and ultimately fatal condiction was associated with diffuse bronchiectasis on CT scan; a 9-year-old girl (patient no. 7), who had a massive, granulomatous pneumonia of the right lung that led to pneumonectomy, and who died after 15 months of bacteriologically ineffective anti–*M. abscessus* treatment; and a 2-1/2-year-old girl (patient no. 8) with segmental condensation of the right mid-lobe on chest x-ray and CT scan, which disappeared only under anti–*M. abscessus* treatment.

**Table:** Chronology of case-patients with *Mycobacterium abcessus* isolation^a^

Case no.	Age (y)/sex	Date of first isolation	No. of pos. cultures/total AFB cultures^b^	No. of pos. AFB smears/total AFB smears^b,c^		*hsp65* genomovar^d^	Sputum microbiologic results^e^
1	2.5 / M	Jan 1996	37/48	19/31	(+++)	-6	Negative
2	13 / F	Feb 1996	2/14	0/9		T	*Pseudomonas aeruginosa*
3	15 / F	Apr 1996	3/41	0/21		-5a	*P. aeruginosa*
4	16 / M	Apr 1996	5/8	1/6	(+)	T	*P. aeruginosa*, *Staphylococcus aureus*, *Aspergillus fumigatus*
5	14 / F	May 1996	8/11	4/8	(++)	T	*P. aeruginosa*, *S. aureus, A. fumigatus*
6	10 / M	May 1996	4/25	0/12		T	*P. aeruginosa, Alcaligenes xylosoxidans*
7	9 / F	May 1996	8/8	4/7	(+++)	T	*P. aeruginosa*, *Aspergillus fumigatus*
8	2.5 / F	Nov 1997	4/6	2/5	(++)	-5a	Negative
9	7 / F	July 1998	2/24	0/7		-5a	*S. aureus*
10	17 / M	Sept 1998	3/9	0/6		T	*S. aureus*, *Haemophilus influenzae*, *Stenotrophomonas maltophilia*, *A. fumigatus*
11	8 / F	Sept 1998	3/12	0/4		T	*P. aeruginosa*, *Staphylococcus aureus*
12	18/F	July 1999	1/5	0/3		T	*P. aeruginosa*, *A. fumigatus*
13	9/M	Sept 1999	1/6	0/3		-6	*S. aureus*
14	16/M	Oct 1999	5/13	4/6	(+)	-5a	*S. aureus*, *A. fumigatus*
15	22/F	Nov 1999	1/6	0/3		-6	*P. aeruginosa*, *S. aureus*

^a^AFB, acid-fast bacilli. ^b^Samples obtained from patients from January 1996 to December 2000; only samples obtained before the administration of antimycobacterial drugs are considered in treated patients. ^c^Symbols in parentheses: AFB density. ^d^See Methods. ^e^Organisms recovered from at least three sputum samples within the 12 months before the first isolation of *M. abscessus*.

All but one (patient no. 1) of the 15 patients were recognized during the study period. Some of the patients who were identified in the first year may have previously gone undetected, as nontuberculous mycobacteria had not been sought before (patients nos. 2, 3, 4, 5, and 7). The *M. abscessus* isolates belonged to genomovars T, -5a, and -6, with a slightly higher prevalence of genomovar T (Table). Genomovar T was involved in the two fatal cases recorded during the study period (patients nos. 6 and 7).

### Characteristics of Patients Positive for *M. abscessus*

The 15 patients positive for *M. abscessus* were predominantly females (sex ratio, 0.40). Their mean age at the time of the first culture positive for *M. abscessus* (11.9 years, range 2.5–22 years) was very similar to the mean age of the entire study population. However, the mean age was lower than that for patients positive for *M. avium* complex (17.5 years; range 13–25 years). Of the 14 patients who underwent genotype analysis, 8 were homozygous for deletion of the phenylalanine in position 508, and 4 were heterozygotous for this deletion plus another mutation. Pulmonary function at the time of the first isolation of *M. abscessus* was highly variable, with forced expiratory volume in 1 second and forced vital capacity values ranging from 14% to 99%, and 31% to 104% of predicted values, respectively. Schwachman score (*20*) also greatly varied among patients (range 40–85). The most prevalent associated disorders included bronchiectasis (13 cases), gastroesophageal reflux (3 cases), and allergic bronchopulmonary aspergillosis (3 cases). All patients had pancreatic insufficiency. Nine of the 15 patients were colonized (at least three positive sputum samples within the previous 12 months) with *Pseudomonas aeruginosa*. None was colonized with *Burkholderia cepacia*.

We analyzed records of all treatments received by the patients within the 12 months preceding the first isolation of *M. abscessus*, including therapeutic aerosols. All of the patients had received IV antibiotics (1–5 two-week IV courses; median 3 courses), combined with aerosol antibiotics at home in 11 patients (tobramycin, 3 patients; colistin, 8 patients). Six patients had received aerosolized deoxyribonuclease. Two patients had received oral corticosteroids, and four had received inhaled corticosteroids.

### Environmental Study

A total of 93 water samples collected from 40 water supply points in the cystic fibrosis center were studied. Three samples (3.2%) from two water supply points tested positive for rapidly growing mycobacteriua (*M. mucogenicum,* two samples; *M. peregrinum*: one sample). None of the samples tested positive for *M. abscessus*. None of the 12 respiratory devices (3 bronchoscopes, 9 nebulizers) studied in October 1997 tested positive for any nontuberculous mycobacteria.

### PFGE Analysis of *M. abscessus* Isolates

PFGE was used to compare the isolates from 14 patients positive for *M. abscessus* (the isolates from patient no. 7 could not be subcultured for testing because of inadequate storage). We studied all isolates from each patient who provided <3 positive cultures and a maximum of five isolates from each patient with >3 isolates. The isolates from three patients (patients nos. 6, 11, and 12) gave unreadable restriction patterns with classical protocols, despite multiple attempts. This phenomenon is common with mycobacteria, particularly *M. abscessus* (*9*) and is probably related to Tris-dependent site-specific cleavage of the DNA (*19*). Nondegradative PFGE was only achieved by running gels in the presence of thiourea, which has been shown to protect the DNA from strand cleavage (*21*). We were therefore able to type all isolates from the 14 patients by PFGE. Each of these 14 patients had isolates of a unique genotype that was unrelated to the genotype of any other patient (Figure). No differences were detected between isolates from the same patient (data not shown), even if the interval between the first and last isolation was as long as 4 years (patient no. 1).

**Figure:**
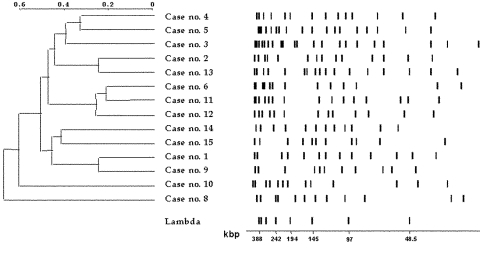
Pulsed-field gel electrophoresis analysis of *Dra*I-digested DNA from *Mycobacterium abscessus* isolates. Restriction patterns of isolates from 14 patients are shown, with a dendrogram of similarity; λ concatemers were used as size standards.

## Discussion

*M. abscessus* was the predominant nontuberculous mycobacterium recovered from the patients attending our center. Approximately 5% of the patientswe screened provided at least one sputum sample positive for this organism, and >65% of these patients had >3 positive samples. Other rapidly growing mycobacteria were far behind (*M. fortuitum*, one patient). This finding confirms that *M. abscessus* differs from *M. chelonae* and from other members of the *M. fortuitum* complex by its particular propensity to cause lung disease in a variety of clinical settings. In a series of 154 cases of lung infection caused by rapidly growing mycobacterium in patients with and without cystic fibrosis, >80% of isolates were *M. abscessus*; *M. fortuitum* was isolated in <15% and *M. chelonae* in <1% of patients (*22*).

In contrast with other studies on cystic fibrosis populations composed of teenagers and adults (*2*,*4*), we found that the *M. avium* complex was isolated less frequently, with an overall prevalence (percentage of patients with at least one positive nontuberculous mycobacterial culture) of approximately 2%. Other pediatric cystic fibrosis centers have reported similar findings (*23*,*24*). This finding suggests that *M. abscessus* is the most prevalent mycobacterial pathogen in children and teenagers with cystic fibrosis. This finding is further supported by the lack of cases involving *M. avium* complex in patients <13 years of age. However, this finding does not preclude epidemiologic variations between countries or institutions.

Nosocomial acquisition of *M. abscessus* has been well documented in patients without cystic fibrosis. Several nosocomial outbreaks of infection or pseudoinfection have been attributed to this organism after cardiac surgery, bacteremia associated with hemodialysis, and pseudoinfections due to contaminated bronchoscopes (*9*). Epidemiologic investigations showed that these outbreaks resulted from the use of contaminated water. Disinfectants may be ineffective against *M. abscessus* in real conditions of use (*12*). PFGE has been used to retrospectively analyze the clinical and environmental isolates recovered during *M. abscessus* outbreaks (*9*). Each of the outbreaks with typeable isolates clearly involved a single strain, which was usually recovered from the water supply system.

Our results do not support nosocomial acquisition of *M. abscessus*. First, the recovery rate of this organism remained constantly low throughout the study. Previous nosocomial outbreaks involving patients without cystic fibrosis were characterized by much higher attack rates. Second, we did not find any link between the use of respiratory devices and the acquisition of *M. abscessus*. Patients positive for *M. abscessus* did not receive more aerosol treatments than did patients with similar clinical status (not shown). The patients used their personal nebulizer at the center and received aerosols in their own rooms. Sterile saline was used when the aerosol was mixed. Reusable respiratory devices were disinfected according to validated protocols and were washed exclusively with sterile water. During the study period, no patients without cystic fibrosis, even severely immunocompromised ones, were infected with *M. abscessus* as a result of a contaminated bronchoscope in our pediatric department. Third, although various rapidly growing mycobacteria were recovered from several water supply points in our center, *M. abscessus* was not isolated. Finally, PFGE analysis demonstrated that the cases involved unrelated strains, which argues against a common source of contamination or patient-to-patient transmission. Similar results have been recently reported with fewer patients (*14*). The low transmissibility, if any, of *M. abscessus* from person to person is further supported by the observation of twin sisters in our series, only one of whom was colonized with *M. abscessus*.

Whether specific measures are necessary to prevent *M. abscessus* infection in patients with cystic fibrosis is questionable (*14*). Our epidemiologic results indicate few potential control approaches exist. A strict segregation policy seems unnecessary because apparently no risk of person-to-person transmission of *M. abscessus* exists (*14*, this study). Further epidemiologic studies are required before recommendations for infection-control precautions can be formulated.
